# Supplementary material for: Indian Biosimilars and Vaccines at Crossroads–Replicating the Success of Pharmagenerics
Source: Vaccines (Basel). 2023 Jan 2;11(1):110. doi: 10.3390/vaccines11010110 (PMC9865573; doi:10.3390/vaccines11010110)
Supplement: Supplementary file 1 [file vaccines-11-00110-s001.zip › vaccines-2043627-supplementary.pdf]

Supplementary materials S1

Replicating success of pharma generics – Indian biosimilars at crossroads

Sunita Panda[1†], Puneet K. Singh[1†], Snehasish Mishra[1†*], Sagnik Mitra[2], Priyabrata Pattnaik[3,4], Sanjib Das Adhikary[5], Ranjan K Mohapatra[6*]

This part briefly describes the major current players in Indian biosimilars market:

1 Indian biopharma R&D major industries and their products

The Indian biopharma sector deals with production and commercialisation of biopharma products like vaccines, therapeutic proteins, drugs and biosimilars for diagnostics and therapeutics, and enjoys a lion's share in global biotechnology market. A few major players are detailed below.

1.1. Biocon

Established in 1978, Biocon is the largest player in the Indian biopharma sector. Based in Bengaluru, Karnataka, Biocon has its presence in 70 countries across the world. It is involved in the production of small molecules, biosimilars, branded formulations and novel molecules. It has subsidiaries like Syngene, Biocon Biologics, BICARA, and Biocon Academy[1].

1.2. Serum Institute of India

Serum Institute of India is the largest vaccine producer globally and considered a top biotech industry in India, founded by Cyrus Poonawalla in 1966 with a vision to produce life-saving immuno-biologicals and make it accessible to the Indian population. Several life-saving biologicals are manufactured and commercialised by it globally, including DTP (Diphtheria-Tetanus-Pertussis) group of vaccines, MMR (Measles-Mumps-Rubella) group of vaccines, tetanus anti-toxin, and anti-snake-venom serum. It is playing a crucial role in producing and distributing Covishield vaccine against the novel corona virus. Serum institute of India is further aiming at producing vaccines for human papillomavirus, meningococcal conjugate vaccine, acellular pertussis vaccine, to name a few [2].

1.3. Santha Biotechnics Ltd, A Sanofi Company

Shantha Biotechnics Ltd, a part of Sanofi group, was established by KI Varaprasad Reddy in 1993. He realised the need of Hepatitis-B vaccine for India at an affordable cost, and decided to develop indigenous vaccine in collaboration with Osmania University in 1993. He then collaborated with the Centre for Cellular and Molecular Biology (CCMB). India's first r-DNA Hepatitis-B vaccine was developed and commercialised successfully by Shantha Biotechnics Ltd in 1997. For his tremendous

effort, Dr. Reddy was awarded the prestigious Padma Bhushan in 2003. A major stake of the company was acquired by Merieux Alliance group of France in November 2006. Then Sanofi, a leading biopharmaceutical industry, took over the company in July 2009[3].

1.4. Biological E

Biological E Ltd was founded in 1953, led by Dr. DVK Raju. Initially, it produced biological products like liver extracts and anticoagulants. Then the company extended its biotechnology wings in 1962 and produced DPT vaccines. Biological E has four strategic business units: branded formulations, speciality generic injectables, synthetic biology and vaccines and biologics. The company collaborates with Baylor College of Medicine, Houston and Dynavax Technologies Corp. to produce Covid-19 vaccine. It aims to produce 100 crore own vaccine dosages and 60 crores more contract manufacturing for the Covid-19 vaccine of Johnson & Johnson this year[4].

1.5. Bharat Biotech

Started by Dr. Krishna M. Ella and Mrs. Suchitra Ella in 1996, Bharat Biotech designs innovative eco-friendly vaccines and biotherapeutics. It is the first biopharma company to develop vaccines against Chikungunya and Zika viruses. It was the first to manufacture recombinant Hepatitis-B vaccine without having cesium chloride and Thiomersal. Attenuated rotavirus vaccine and attenuated strain, and typhoid conjugate vaccine are also Bharat Biotech products. Its other product is REGEN-D, a popular therapeutic epidermal growth factor for diabetic foot ulcers and burns. It developed Covaxin as the first completely indigenous Covid-19 vaccine in collaboration with ICMR and NIV. The vaccine has got DCGI approval for emergency administration[5].

1.6. Panacea Biotec

First set up in 1984 as Panacea Drugs Pvt Ltd, it extended a unit named 'Radicura Pharma' in 1988 to produce vaccine. Both the units were merged in 1993 to form Panacea Biotec Ltd. The company focuses to produce therapeutics and vaccines for certain symptoms such as pain, diabetes, cardiovascular diseases, oncology, renal disease, tuberculosis, osteoporosis and gastrointestinal problems. It is one of the largest vaccines producing companies in India and it is committed for full range oral polio vaccine production. It collaborates with National Institute of Immunology (NII) for vaccine against Japanese encephalitis, and also tied with BCIL to produce and distribute Anthrax vaccine worldwide. Also, process to manufacture Sputnik V vaccine against novel corona virus under technology transfer from Russian Direct Investment Fund (RFID) is underway[6].

1.7. Dr Reddy's Laboratories Pvt Ltd

Established in 1984 by Dr. K. Reddy with a vision to make easy accessibility of drugs to everyone, it diversified to the production of generics and APIs from 2007. It started biosimilars business with the production of Rituximab to treat non-Hodgkin's lymphoma. Upon successful completion of clinical trials demonstrating the effectiveness of Sputnik V vaccine for Covid-19, the company awaits DCGI approval for its emergency use (41). Institute of Nuclear Medicine and Allied Sciences, a lab of DRDO in collaborations with Dr Reddy's Laboratories has developed an oral antiviral drug, 2-Deoxy-D-Glucose (2DG), approved by DCGI on 1st May 2021. This is an adjunct therapy for moderate to severe COVID-19 patients[7].

1.8. Reliance Life Science Pvt Ltd

A subsidiary of the Reliance Group of Industries, the organisation provides business opportunities for effective biotherapeutics like plasma proteins, biosimilars, monoclonal ABs, stem cell therapies, molecular medicines, oncology generics and clinical research services. It produces biosimilars for a wide range of therapeutic areas such as cancer, cardiac manifestation, rheumatoid arthritis, diabetes, autoimmune diseases, female infertility, etc. It has the largest number of biosimilars in Indian market, and globally has the highest number of biosimilars at research and development stages. It has currently initiated the marketing of biotherapeutics overseas[8].

1.9. Zydus Cadila

Established in 1952 by late Ramanbhai B. Patel with a vision to provide innovative solutions to unmet healthcare problems, the company provides a wide range of therapeutic in generics, vaccines and biologics. Adalimumab, world's first biosimilar for inflammatory rheumatoid arthritis, is a Zydus Cadila product. It has developed Twin RABs, the first novel cocktail of antirabies monoclonal antibodies. Vaxiflu 4, India's first tetravalent inactivated influenza vaccine, was developed by the Zydus Cadila group which provides immunity against four strains, *viz.*, H1N1, H3N2, Type B (Brisbane) and Type B (Phuket). It has a vaccine technology centre that has developed vaccines against pertusis, tetanus, *Haemophilus influnzae*, Hepatitis B, typhoid, mumps, Rubella. Vaccine research in the centre is underway for human papilloma virus, Ebola, leishmaniasis, Japanese encephalitis, etc.[9].

1.10. Indian Immunologicals

It is the market leader in veterinary and human biologicals in India. In 1982, National Dairy Development Board (NDDB) established Indian immunologicals as its unit to provide vaccines for veterinary use in an affordable rate. Then this unit was incorporated as Indian Immunologicals Limited in 1999. Indian Immunological Limited is the first Indian company and second in the world to produce Purified Vero cell rabies vaccines, 'Abhayrab'. It manufactures and promotes more than

150 products such as vaccines, hormones and biogenerics. It operates the largest veterinary vaccine unit in the world. The company has a pipeline of potential viral vaccines including Zika, dengue, varicella, and COVID-19. Indian immunologicals in partnership with Griffith University in Australia, developed a potential live attenuated vaccine candidate against COVID-19 by using codon deoptimization technology. They are stating it would be safe, active and provide a prolonged immunity against COVID-19 even in a single immunisation dose. It is also now partnered with Bharat Biotech to manufacture the drug substance for COVAXIN® through technology transfer and its underway[10].

References

1. Biocon - India's largest biopharmaceutical company [Internet]. [cited 2022 Sep 4]; Available from: https://archive.biocon.com/
2. Serum Institute of India | Manufacturer of Vaccines & immuno-biologicals - GMP Vaccine Manufacturer [Internet]. [cited 2022 Sep 4]; Available from: https://www.seruminstitute.com/
3. Chakma J, Masum H, Perampaladas K, Heys J, Singer PA. Indian vaccine innovation: The case of Shantha Biotechnics. Global Health 2011; 7.
4. Biological E Ltd [Internet]. [cited 2022 Sep 4]; Available from: https://biologicale.com/
5. History & Milestones - Bharat Biotech - A Leading Biotech Company [Internet]. [cited 2022 Sep 4]; Available from: https://www.bharatbiotech.com/history_milestones.html
6. Panacea Biotec [Internet]. [cited 2022 Sep 4]; Available from: https://www.panaceabiotec.com/en
7. Dr. Reddy's Laboratories - Multinational Pharmaceutical Company [Internet]. [cited 2022 Sep 4]; Available from: https://www.drreddys.com/
8. Clinical Research Services, Regenerative Medicine, Specialty Pharmaceuticals | Reliance Life Sciences - LifeSciences [Internet]. [cited 2022 Sep 4]; Available from: https://rellife.com/
9. Zydus pharma Research Center | Vaccine Technology Centre In India [Internet]. [cited 2022 Sep 4]; Available from: https://www.zyduslife.com/research
10. Indian Immunologicals Ltd (IIL) | Leading Animal & Human Vaccine Manufacturer - IIL [Internet]. [cited 2022 Sep 4]; Available from: https://www.indimmune.com/

2. Government and private initiatives

Indian government is proactively promoting biologics and biosimilars under 'Make in India' and 'Start-up India' campaigns. These initiatives focus on active industry-academia collaboration, encourage investments, promote entrepreneurship, initiate bioindustry start-ups and empowering human resources. 'National Bio-Pharma Mission', a DBT-World Bank joint initiative worth US$ 250 Million accelerates the progress of innovative biopharmaceuticals at early stage. This mission has supported the development of biotherapeutics like Insulin Glargine from Vitane Pharmaceuticals, Herceptin from Serum Institute of India, and Plasma fractionation[1,2] (BIBCOL), MynVax, Pneutgar-15, TV003/TV005, DSV4 and DSV4+2E VLP .

2.1. Biotechnology Industry Research and Development Assistance Council (BIRAC)

The Department of Biotechnology (DBT) established BIRAC to boost up industry-academia link to support small and medium bioenterprises and start-ups, and to promote public-private partnerships. BIRAC helps in facilitating industrial research through new technologies, their validation, transfer and intellectual property management. It promotes and facilitates innovative research and commercialisation of innovative discoveries in key social sectors. BIRAC has supported 801 start-ups and has enabled 52 bio-incubators. BIRAC as established by DBT as a not-for-profit public sector enterprise has financially supported more than 500 companies and nearly 800 start-ups. BIRAC provides national and global network opportunity. In addition, it strengthens public-private partnerships and industry-academia partnerships for translational research and industrial growth in Indian subcontinent BIRAC floats several schemes from time to time to promote research and development in vaccines, biosimilars, regenerative medicines, diagnostics, agriculture, clean energy and the environment[3,4], a few of which are detailed below.

2.1.1. Biotechnology Ignition Grant (BIG)

The BIG scheme supports young start-ups and entrepreneurs by providing a one-time grant of Rs.50 lakh to establish proof-of-concept having translational potential. Rs. 275 crores of funds have been invested so far supporting 550 proposals and 150 start-ups. BIG has supported 140 women entrepreneurs in developing 50 innovative products and generating 175 IPs[5,6].

2.1.2. Small Business Innovation Research Industry (SIBRI)

Launched in 2005, SIBRI supports early-stage funding for high-risk innovative research to develop products. Existing small and medium bioenterprises planning to expand are also funded. It covers all expenses from early stage of product development till commercialisation. SIBRI has funded Rs. 272 crore in 295 projects, developed and commercialised 56 products and generated 35 patents (31). The small preliminary projects are approved by SIBRI, while the bigger projects would fit into BIPP. SIBRI encourages small innovative projects while BIPP is for grander projects[7].

2.1.3. Biotechnology Industry Partnership Programme (BIPP)

Launched on 5th Dec 2008 by the DBT, the BIPP is a highly industry-friendly initiative that has approved 214 proposals worth Rs. 523.79 crore. BIPP is a late-phase Industry-BIRAC collaborative funding that helps in scaling and commercialising high-risk innovations[8].

2.1.4. Promoting Academic Research Conversion to Enterprise (PACE)

PACE is an industry-academia partnership interface programme. It is divided into two components, the Academic Innovation Research that supports academic researchers to develop a process or product up to a proof-of-concept stage, and the Contract Research Scheme which supports the validation of techniques or products developed by academics by industry partners. PACE has invested Rs. 83.84 crore in 105 projects with 58 collaborations, successfully generating seven products and five patents[9].

2.1.5. Bio Nest Scheme

Earlier known as Bioincubator supporting system (BISS), it supports bioincubators providing appropriate infrastructure and high-end instrumentation to the entrepreneurs and start–ups. It also provides a platform for networking, IP and regulatory guidance. There are 50 such bioincubators established in academic and research institute, or in medical schools and hospitals across the country, additionally supported by private sector, and/or the central and state governments[10].

2.1.6. Equity Funding Scheme

A sizable financial support is needed for a start-up particularly during the initial phase. This scheme supports selected promising bioincubator start-ups having the potential for translational growth. It also helps such start-ups to attract investments from other investors like angel investors and venture capitalists[11]. It supports a proof-of-concept proposal to its commercialisation in three phases as below.

2.1.6.1. ***Sustainable entrepreneurship and enterprise development fund (SEED Fund)*** – The grant releases up to 30 lakhs initially to a few selected early-stage start-ups at proof-of-concept phase.

2.1.6.2. ***Launching entrepreneurial driven affordable product fund*** – Up to 100 lakh is funded to the potential start-ups to upgrade the operation to the pilot-scale stage or the pre-commercialisation stage. Such funds are provided to the bioincubators having SEED and LEAP partners.

2.1.6.3. ***BIRAC's Accelerating Entrepreneur Fund*** – It provides funds to biotech start-ups during the product development and growth phase. The funds are amalgamated with SEBI registered venture funds.

2.2. Impacting Research Innovation and Technology Project (IMPRINT)

The Department of Science and Technology (DST) in association with the Ministry of Human Resource Development (MHRD) implements the IMPRINT scheme to promote research and innovations in social fronts like healthcare, energy, sustainable habitat, water resources, advance material science, nanotechnology and on environment and climate studies. It is funded through the SERB to the institutions with more demand-driven development solution. Its subsidiary scheme IMPRINT2 targets translational research, product development and implementation in challenging areas[12].

2.3. Association of Biotechnology Led Enterprises (ABLE)

Representing the Indian biotechnology sector, the ABLE was launched in April 2003 as a not-for-profit organisation. The primary focus of this forum is to promote the growth of biotechnology, to create optimal policies and regulatory environment to encourage entrepreneurship and investments in India through government partnerships. It promotes both domestic and overseas collaborations and partnerships[13].

References:

1. Burnouf T. Modern Plasma Fractionation. Transfus Med Rev [Internet] 2007 [cited 2022 Sep 7]; 21:101. Available from: /pmc/articles/PMC7125842/

2. BIRAC [Internet]. [cited 2022 Sep 4]; Available from: https://birac.nic.in/big.php

3. BIRAC-Home [Internet]. [cited 2022 Sep 4]; Available from: https://www.birac.nic.in/

4. BIRAC-Biotechnology Ignition Grant (BIG) Scheme [Internet]. Available from: www.birac.nic.in

5. Biotechnology Industry Research Assistance Council (BIRAC) A Government of India Enterprise Biotechnology Ignition Grant (BIG) Igniting New Ideas….. Scheme Guidelines

6. BIRAC-New [Internet]. [cited 2022 Sep 7]; Available from: https://birac.nic.in/desc_new.php?id=217

7. BIRAC-New [Internet]. [cited 2022 Sep 7]; Available from: https://birac.nic.in/desc_new.php?id=216

8. BIRAC-New [Internet]. [cited 2022 Sep 7]; Available from: https://birac.nic.in/desc_new.php?id=286

9. BIRAC-BioNEST [Internet]. [cited 2022 Sep 7]; Available from: https://birac.nic.in/bionest.php

10. What are Equity Mutual Funds? Meaning, Types, Benefits, Return & Taxation [Internet]. [cited 2022 Sep 7]; Available from: https://www.sbimf.com/en-us/what-are-equity-mutual-funds

11. IMPRINT India Initiative [Internet]. [cited 2022 Sep 7]; Available from: https://imprint-india.org/

12. Association of Biotechnology Led Enterprises [Internet]. [cited 2022 Sep 7]; Available from: https://www.ableindia.in/
